# Decreasing incidence of *Plasmodium vivax* in the Republic of Korea during 2010–2012

**DOI:** 10.1186/1475-2875-12-309

**Published:** 2013-09-05

**Authors:** Tong-Soo Kim, Jin Su Kim, Byoung-Kuk Na, Won-Ja Lee, Heung-Chul Kim, Seung-Ki Youn, Jin Gwack, Hee Sung Kim, PyoYun Cho, Seong Kyu Ahn, Seok Ho Cha, Yun-Kyu Park, Sung Keun Lee, Yoon-Joong Kang, Youngjoo Sohn, Yeongseon Hong, Hyeong-Woo Lee

**Affiliations:** 1 Departments of Parasitology, Inha University School of Medicine, Incheon 400-712, Republic of Korea; 2 Department of Parasitology and Institute of Health Sciences, Gyeongsang National University School of Medicine, Jinju 660-751, Republic of Korea; 3 Division of Malaria and Parasitic Diseases, National Institute of Health, Osong 363-951, Republic of Korea; 4 5th Medical Detachment, 168th Multifunctional Medical Battalion, 65th Medical Brigade, Unit 15247, APO AP 96205-5247, USA; 5 Department of Epidemiology, National Institute of Health, Osong 363-951, Republic of Korea; 6 Department of Biomedical Technology, Inha University School of Medicine, Incheon 400-712, Republic of Korea; 7 Department of Pharmacology, Inha University School of Medicine, Incheon 400-712, Republic of Korea; 8 Department of Biomedical Science, Jungwon University, Goesan, Chungbuk 367-805, Republic of Korea; 9 Department of Anatomy, College of Korean Medicine, Institute of Korean Medicine, Kyung Hee University, Seoul 130-701, Republic of Korea; 10 Department of Public Health, Sahmyook University, Seoul 139-742, Republic of Korea; 11 Department of Pathology, Immunology, and Laboratory Medicine, College of Medicine, University of Florida, J-566, 1275 Center Drive, Gainesville, FL 32610, USA

## Abstract

**Background:**

After the re-emergence of *Plasmodium vivax* in 1993, a total of 31,254 cases of vivax malaria were reported between 1993–2012 in the Republic of Korea (ROK). The purpose of this study was to review Korea Centers for Disease Control and Prevention records to investigate the transmission of malaria from 2010–2012.

**Methods:**

Reporting of microscopy-diagnosed cases of malaria is mandatory in the ROK. In this study, all available records of malaria cases and malaria vectors collected from 2010 – 2012 in Cheorwon County, Gangwon Province and Ganghwa County, Incheon Metropolitan City, were reviewed.

**Results:**

Although the number of cases of malaria peaked a third time in 2010 (1,772 cases) since the re-emergence of *P. vivax*, the incidence decreased two-fold to 838 in 2011 and three-fold to 555 in 2012. The number of cases decreased 52.7% in 2011 compared with that in 2010 and 33.8% in 2012 compared with that in 2011. However, the number of cases increased in Incheon Metropolitan City (15.3%) and Gyeongnam Province (23.1%) in 2012 compared with 2011. Of the 3,165 cases of vivax malaria in 2010–2012, 798 (25.2%) were in ROK military personnel, 519 (16.4%) in veterans, and 1,848 (58.4%) in civilians. In total, there were 2,666 male patients and 499 female patients, and the ratio of female to male patients increased from 1:7.9 in 2011 to 1:4.1 in 2012.

**Conclusions:**

A rapid decrease in the incidence of malaria was observed in most areas from 2010 to 2012, but the incidence increased again in the western part of the demilitarized zone. Therefore, more intensive surveillance is needed throughout high risk areas to identify factors responsible for increase/decrease in the incidence of malaria in the ROK.

## Background

*Plasmodium vivax* is the causative agent of relapsing benign tertian human malaria, the second most common type of malaria in humans, that afflicts several hundred million people annually in the world. Vivax malaria is a major public health problem in many tropical and semi-tropical regions and temperate countries, including the Democratic People’s Republic of Korea (DPRK) and the Republic of Korea (ROK) [1].

The first scientific documentation of malaria was published in 1913, although it had been prevalent throughout the Korean peninsula for several centuries [2]. As a result of a national malaria eradication programme conducted in cooperation with the World Health Organization (WHO), the incidence of vivax malaria in the ROK rapidly decreased until in 1979 when WHO declared the ROK to be malaria free [3-5]. In the 1980’s two sporadic cases were detected [6] and in 1993, a ROK soldier serving in northern Gyeonggi Province [7], and two civilians were diagnosed with vivax malaria [8]. Thereafter, a number of malaria cases were reported near the demilitarized zone (DMZ), which centers on Paju-si, Yeoncheon-gun, Cheorwon-gun, Gimpo-si, Ganghwa-gun, Goyang-si, and Dongducheon-si.

During the first few years of re-emergence of vivax malaria in the ROK, most cases occurred in ROK military personnel deployed near the DMZ. But some civilians who live in Daeseongdong where located inside of DMZ and Tongilchon surrounded by ROK military installations near the DMZ showed high infection rate as much as ROK military personnel. In addition, as the number of malaria cases increased, the ratio of civilian cases were increased [9].

Thus, it was of considerable concern that vivax malaria might become re-established through out the ROK as veteran soldiers returned to their hometowns throughout all of Korea [10-12].

The aim of this study was to determine the annual number of patients with malaria among military personnel, veterans, and civilians in the ROK; the mean daily incidence for weekly intervals; the geographic distribution of cases among military personnel and civilians; and the number of *Anopheles* mosquitoes captured by black light trap in the malaria risk from 2010–2012 to analyse the current status of malaria in the ROK.

## Methods

### Ethics statement

All participants were informed about the study methodology and provided written informed consent according to ethical standards. The study procedures, potential risks, and benefits were explained to all participants. All adult participants and parents/guardians in the household of participants under 18 years of age provided informed consent. Parents who were unwilling to have their children participate in the study were identified and their children subsequently excluded without prejudice from study surveys. All patient data were anonymous. The Human Ethics Committee of Inha University provided ethical approval, and this study was conducted according to the principles expressed in the Declaration of Helsinki.

### Data collection from patients with malaria

Malaria is classified as one of the Group III communicable diseases that should be controlled by the Korean government. For the convenience of data analysis, patients with malaria were categorized as civilians, veterans, and military personnel. For civilians and veterans (who were diagnosed with vivax malaria within 24 months of discharge from military service in malaria epidemic areas), cases of malaria detected in private hospitals or clinics are reported to the local Public Health Center (PHC). The data collected by the PHCs is provided periodically to the Division of Infectious Disease Surveillance, Korea Centers for Disease Control and Prevention. For military personnel, cases of malaria are reported to the Office of the Surgeon General, Army Headquarters, and the Ministry of Health and Welfare [13]. The report usually contains the patient’s name, age, sex, address, date of onset of illness, date of diagnosis of malaria, and estimated areas where possibly infected. Most cases of malaria diagnosed in civilian hospital or health clinics include veterans, but might also include ROK soldiers who were diagnosed while leave. However, these cases among ROK soldiers are excluded from the civilian/veteran populations by the Division of Infectious Disease Surveillance, since the patients are contacted directly as part of the verification process [14,15].

### Definition of malaria

Thick and thin blood films obtained from suspected malaria patients were fixed with methanol and stained with Giemsa diluted with buffered water at pH 7.2 in accordance with standard procedure [16]. To estimate the densities of blood-stage parasites, the numbers of parasites were counted relative to 200 white blood cells (WBCs) and then the parasite/WBC ratio was multiplied by 8,000 (estimated number of WBCs per microlitre of blood) [17].

### Geographic distribution of patients with malaria

The annual geographic distribution of *P. vivax* malaria in civilians, veterans, and ROK military personnel was determined by the patients residence and travel history during diagnosis. The seasonal incidence was analysed by grouping cases at weekly or monthly intervals.

### Calculation of the annual parasite incidence

The annual parasite incidence (API) was calculated as the number of malaria-positive patients per 1,000 inhabitants for each of the study sites: API = (number of microscopically proven malaria cases/1,000 population/year).

### Density of *Anopheles* mosquitoes

**Figure 1:**
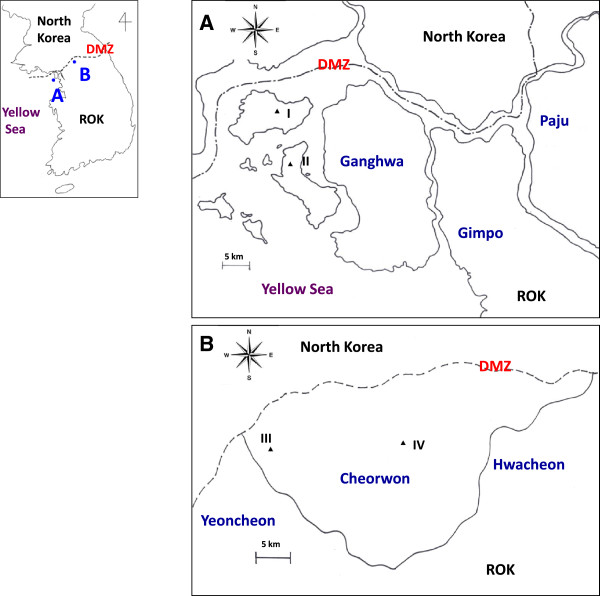
**Locations where collected mosquitoes by black light traps. A**, Ganghwa County; **B**, Cheorwon County. Mosquito collection area: **I**, Gyodong-myeon; **II**, Samsan-myeon; **III**, Cheorwon-eup; **IV**, Gimhwa-eup.

The number of *Anopheles* mosquitoes was monitored using a black light traps in Gyodong-myeon (Figure 1A, I) and Samsan-myeon (Figure 1A, II), Ganghwa County of Incheon Metropolitan City and Cheorwon-eup (Figure 1B, III) and Gimhwa-eup (Figure 1B, IV), Cheorwon County of Gangwon Province in the ROK during the malaria transmission season from 2010–2012. Mosquito collections were conducted twice a week between 8 PM and 7 AM from April - October, at the same collection sites for comparative analysis. Female mosquitoes were identified using morphological differences and counted regardless of the blood-fed status or malaria parasite infectivity. The average of the number of collected mosquitoes over two days was considered a representative value for the week [14].

## Results

### Overview of malaria in the past two decades

**Figure 2:**
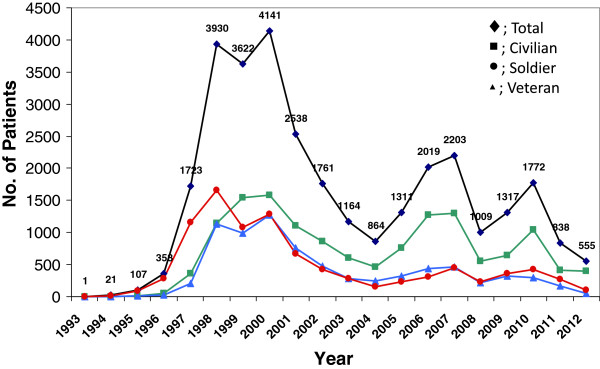
The annual number of vivax malaria cases from 1993–2012.

A total of 31,254 cases of vivax malaria were reported from 1993–2012 (Figure 2). In total, 9,501 cases (30.4%) were reported in ROK military soldiers, 7,658 cases (24.5%) in veterans who served in malaria risk areas, and 14,095 cases (45.1%) in civilians during the 20 years after the re-emergence of malaria in the ROK. There were three peaks in the incidence of vivax malaria during last two decades, with 4,141 cases, 2,203 cases, and 1,772 cases in 2000, 2007, and 2010, respectively.

### Patients with malaria from 2010–2012

**Figure 3:**
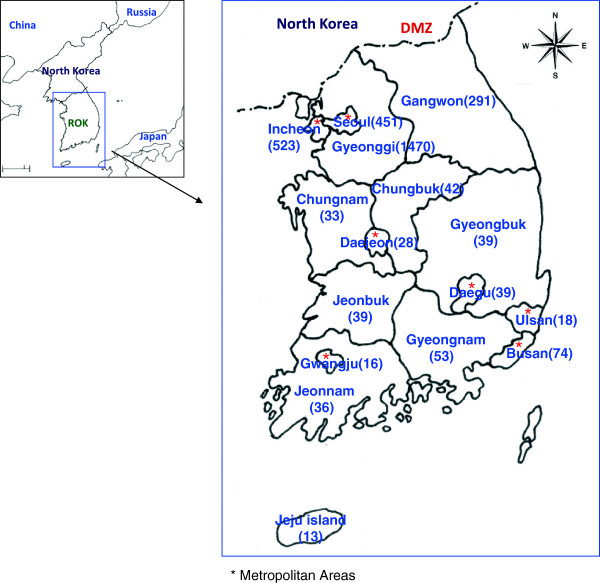
**Reported vivax malaria cases in the Republic of Korea (ROK) by Province and Metropolitan Area.** DMZ, Demilitarized Zone.

A total of 3,165 vivax malaria cases were reported from 2010–2012, including 798 (25.2%) in soldiers, 519 (16.4%) in veterans (less than 2 years after active duty service), and 1,848 (58.4%) in civilians (Table 1). The proportion of annual number of civilian cases increased from 48.6% in 2011 to 71.0% in 2012, while the proportion of ROK soldiers and veterans decreased from 264 (31.5%) in 2011 to 104 (18.7%) in 2012 and from 167 (19.9%) in 2011 to 57 (10.3%) in 2012, respectively. Overall, the annual proportion of malaria cases for 16 administrative districts decreased 52.7% in 2011 compared to 2010 and by 33.8% compared to 2011. However, the number of cases reported from the Incheon Metropolitan City and Gyeongnam Province (Figure 3) increased 15.3% and 23.1%, respectively, in 2012 (Table 2). In addition, the US military reported a total of nine malaria cases during 2010–2012. Six cases were reported while deployed to Korea (4 cases in 2010, 1 case in 2011, and 1 case in 2012), while three cases were reported after returning to the USA (due to the long-incubation period of vivax malaria, one case in 2010, 0 case in 2011, and two cases in 2012).

**Table 1:** **Annual number (percent of total) of *Plasmodium vivax *cases among ROK military personnel, veterans, and civilians**

	**2010**	**2011**	**2012**	**Total**
Veteran	295 (16.6%)	167 (19.9%)	57 (10.3%)	519 (16.4%)
Soldier	430 (24.3%)	264 (31.5%)	104 (18.7%)	798 (25.2%)
Civilian	1047 (59.1%)	407 (48.6%)	394 (71.0%)	1848 (58.4%)
Total	1772 (56.0%)	838 (26.5%)	555 (17.5%)	3165

**Table 2:** **Annual number and percent difference of *Plasmodium vivax *cases by Metropolitan City and Province, 2010-2012**

	**No. cases 2010**	**No. cases 2011**	**Percent difference 2011**	**No. cases 2012**	**Percent difference 2012**
**Metropolitan areas**					
Seoul	290	94	-67.6	67	-28.7
Busan	43	24	-44.2	7	-70.8
Daegu	19	16	-15.8	4	-75.0
Incheon	256	124	-51.6	143	+15.3
Gwangju	9	6	-33.3	1	-83.3
Daejeon	15	9	-40.0	4	-55.6
Ulsan	8	7	-12.5	3	-57.1
**Provinces**					
Gyeonggi	818	389	-52.4	263	-32.4
Gangwon	184	94	-48.9	13	-86.2
Chungbuk	23	11	-52.2	8	-27.3
Chungnam	14	12	-14.3	7	-41.7
Jeonbuk	22	12	-45.5	5	-58.3
Jeonnam	21	11	-47.6	4	-63.6
Gyeongbuk	19	12	-36.8	8	-33.3
Gyeongnam	24	13	-45.8	16	+23.1
Jeju	7	4	-42.9	2	-50.0
Total	1772	838	-52.7	555	-33.8

### Comparison of the API according to administrative areas

Overall, API decreased from 0.036 in 2010 to 0.017 in 2011 and 0.011 in 2012 (Table 3). In 2010, Gangwon Province had the highest API at 0.123, followed by Incheon Metropolitan City (0.093), Gyeonggi Province (0.069), and Seoul Metropolitan City (0.029). In 2011, Gangwon Province had the highest API at 0.063, followed by Incheon Metropolitan City (0.045), Gyeonggi Province (0.033), and Seoul Metropolitan City (0.009). In 2012, Incheon Metropolitan City had the highest API at 0.052, followed by Gyeonggi Province (0.022), Gangwon Province (0.009), and Seoul Metropolitan City (0.007).

**Table 3:** **The number of *Plasmodium vivax *malaria patients and annual parasite incidence from 2010-21012 by Metropolitan City and Province**

		**2010**	**2011**	**2012**
	**No. of population**	**No. of patient (API)**	**No. of patient (API)**	**No. of patient (API)**
**Metropolitan areas**				
Seoul	10,026,000	290 (0.029)	94 (0.009)	67 (0.007)
Busan	3,464,000	43 (0.012)	24 (0.007)	7 (0.002)
Daegu	2,477,000	19 (0.008)	16 (0.006)	4 (0.002)
Incheon	2,750,000	256 (0.093)	124 (0.045)	143 (0.052)
Gwangju	1,506,000	9 (0.006)	6 (0.004)	1 (0.001)
Daejeon	1,527,000	15 (0.010)	9 (0.006)	4 (0.003)
Ulsan	1,105,000	8 (0.007)	7 (0.006)	3 (0.003)
**Provinces**				
Gyeonggi	11,788,000	818 (0.069)	389 (0.033)	263 (0.022)
Gangwon	1,496,000	184 (0.123)	94 (0.063)	13 (0.009)
Chungbuk	1,539,000	23 (0.015)	11 (0.007)	8 (0.005)
Chungnam	2,104,000	14 (0.007)	12 (0.006)	7 (0.003)
Jeonbuk	1,802,000	22 (0.012)	12 (0.007)	5 (0.003)
Jeonnam	1,772,000	21 (0.012)	11 (0.006)	4 (0.002)
Gyeongbuk	2,638,000	19 (0.007)	12 (0.005)	8 (0.003)
Gyeongnam	3,232,000	24 (0.007)	13 (0.004)	16 (0.005)
Jeju	552,000	7 (0.013)	4 (0.007)	2 (0.004)
Total	49,778,000	1,772 (0.036)	838 (0.017)	555 (0.011)

**Figure 4:**
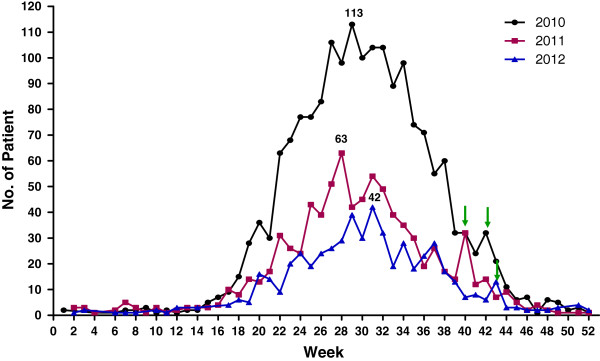
The weekly incidence of vivax malaria in ROK patients from 2010–2012.

Most cases of malaria were reported from June through September in 2010–2012 with the highest peaks observed from the 29th week of 2010 (July 10–17) with 113 patients, in the 28th week of 2011 (July 2–9) with 63 patients, and in the 31st week of 2012 (July 28 to August 4) with 42 patients (Figure 4). Interestingly, the last apparent peaks of annual incidence during these three years were approximately three weeks after Chuseok (Korean Thanksgiving Day, which was on September 22 in 2010, September 12 in 2011, and September 30 in 2012), that is, the 42nd week of 2010 (October 10–16) with 32 patients, the 40th week of 2011 (October 2–8) with 32 patients, and the 43rd week of 2012 (October 21–27) with 13 patients (Figure 4).

### Sex ratio and occupation of vivax malaria patient

**Figure 5:**
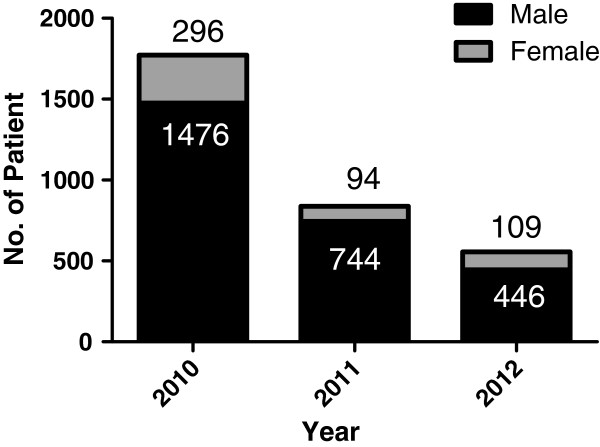
The sex ratio of patients with vivax malaria from 2010–2012.

**Figure 6:**
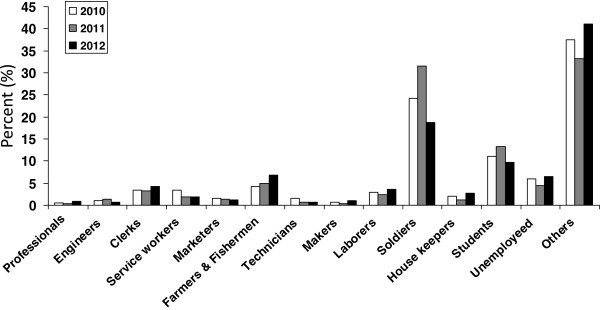
Occupations of vivax malaria patients from 2010–2012.

There were 2,666 male and 499 female patients with vivax malaria from 2010–2012. The ratio of female to male patients increased from 1:7.9 in 2011 to 1:4.1 in 2012 (Figure 5). The most common occupation was soldier (18.74–31.50%), followed by students (9.73–13.25%), and farmers and fishermen (4.23–6.85%) (Figure 6).

### Density of *Anopheles* mosquitoes

**Figure 7:**
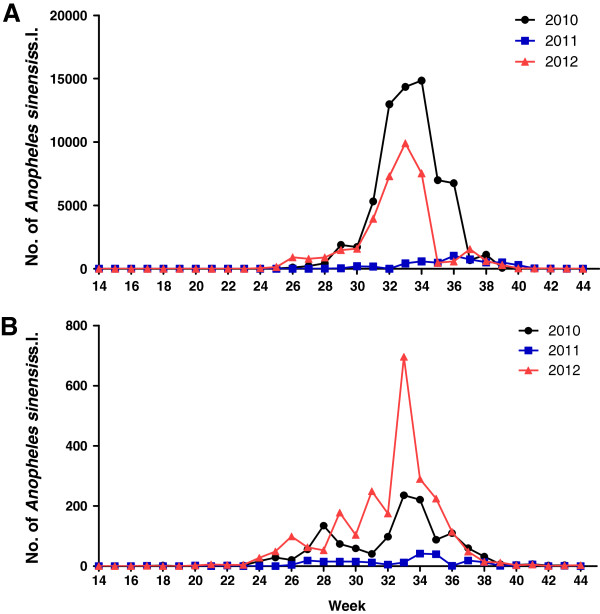
**The weekly number of *Anopheles sinensis *s.l. in Ganghwa County, Incheon Metropolitan City (A) and Cheorwon County, Gangwon Province (B) from 2010–2012.**

A total of 67,566 *An. sinensis* sensu lato were collected in 2010, 5,022 in 2011, and 38,207 in 2010 in Ganghwa County from April - October (Figure 7A). In Cheorwon County, 1,308 *An. sinensis* s.l. were collected in 2010, 228 in 2011, and 2,427 in 2010 (Figure 7B). *An. sinensis* s.l. was first detected during the 17th week in Cheorwon County in all three years; however, in Ganghwa County, *An. sinensis* s.l. was first detected during the 17th week of 2010, 18th week of 2011, and 15th week of 2012 (Figure 7).

The highest density of *An. sinensis* s.l. was recorded during the 33rd week (236) of 2010, the 34th week (42) of 2011, and the 33rd week (696) of 2012 in Cheorwon County. In Ganghwa County, the highest density of *An. sinensis* s.l. was reported during the 34th week (14,848) of 2010, the 36th week (1,027) of 2011, and the 33rd week (9,909) of 2012. The number of *An. sinensis* s.l. was lowest at the end of the surveillance period (Figure 7).

A total of 110,795 *An. sinensis* s.l. were collected in Ganghwa County but only 3,963 were collected in Cheorwon County (Figure 7). The high vector density in Ganghwa County may have resulted in the high and increased incidence of vivax malaria in that area.

## Discussion

In North Korea, the high-risk areas of vivax malaria are Gaeseong City, the South and North Hwanghae Provinces, and Gangwon Province, which border the DMZ. The DMZ is a 4-km-wide, 250-km-long corridor that extends across the middle part of the Korean peninsula. In general, no civilians have been allowed to enter the DMZ for more than 60 years, even though one exceptional village called “Daesungdong” is located inside of DMZ; therefore, natural ecosystems and biodiversity are highly conserved in the DMZ [18]. To control malaria, North Korea has provided presumptive anti-relapse chemoprophylaxis with primaquine (15 mg for 14 days) before the malaria transmission season for civilians living in these areas since 2002 [19]. As a result, it was believed that transmission of malaria occurred mainly in these areas in North Korea [20]. Therefore, the adjacent to malaria risk areas in North Korea, high risk areas were observed in the ROK, e.g., Incheon Metropolitan City and northern Gyeonggi and Gangwon provinces. There is no objective opinion that mass chemoprophylaxis might largely decrease the number of malaria cases among military personnel, which may alternatively decrease numbers of cases among civilian populations. Chemoprophylaxis started with approximately 16,000 military personnel in 1997 and expanded to more than 200,000 military personnel who are currently serving in malaria high risk areas [13]. Unfortunately, it was found that prophylactic failure occurred even in those who had sufficient plasma concentrations of hydroxychloroquine. It means that a chloroquine-resistant *P. vivax* strain has been reported in the ROK [21]. The appearance of this drug-resistant strain is troublesome for the treatment of patients in the future. Therefore, another standard regimen should be considered to replace the current regimen of chloroquine-primaquine. However, it is not easy to find the next defensive line of anti-malaria drugs because it has been reported that Korean *P. vivax* isolates are already resistant to pyrimethamine, which is recommended for use in patients with malaria who fail to respond to chloroquine chemotheraphy [22]. It was shown that the existence of antifolate-resistant *P. vivax* in the ROK. Further detailed geographic mapping of current and changing patterns of vivax malaria drug resistance on a national or regional scale would prove a valuable aid for developing and updating national anti-malarial policy guidelines in the ROK. Control measures and inter-governmental co-operation are also needed to block the spread of drug-resistant malaria in the ROK.

It was reported that six Anopheles species in the ROK: *Anopheles sinensis* sensu stricto*, Anopheles lesteri, Anopheles pullus, Anopheles sineroides, Anopheles kleini*, and *Anopheles belenrae*[23-26]. These six species comprise a species complex called *An. sinensis* s.l. Because they occur in sympatry and it is hard to distinguish by morphology. Thus, molecular methods are needed to identify the species. But it is hard to apply the newly developed methods for classifications in the surveillance system until developing more precise methods for identification of *Anopheles* complex in real time manners. Therefore, *An. sinensis* s.l. was presented in this study. Analysis of the mosquito density of *An. sinensis* s.l. in study areas over three years showed an extremely higher density in Ganghwa County compared to Cheorwon County (Figure 7). Therefore, the potential unknown threatening factors including mosquito density still may exist on the west side of malaria foci compared with the east side; this explains why the API of Gangwon Province, which is the representative location of the east side, was reduced dramatically from 0.123 in 2010 to 0.063 in 2011 and to 0.009 in 2012 and the API of Incheon Metropolitan City changed from 0.093 in 2010 to 0.045 in 2011 but increased to 0.052 in 2012 (Table 3).

PHCs are responsible for controlling the transmission of malaria in each of the vivax malaria risk area. As a result of the anti-malaria efforts of the PHC in Cheorwon County, which is a high-risk area in Gangwon Province, the number of cases of malaria decreased dramatically to low level, 27 cases in 2010, 14 cases in 2011, and two cases in 2012. In Cheorwon County, nine cases were first reported in 1997, while there was a peak of 166 cases in 2000 [27]. It took an average of 5.4 average days (4.2–7.0) to confirm a case of malaria after onset during 2010–2012 (unpublished data). Anti-mosquito activities, including fogging and residual spray, took place from April 8 to October 5 in 2010, May 4 to October 4 in 2011, and May 4 to October 9 in 2012. In total, 748 L of insecticide and 129 L of larvicide were used in 2010, 1,037 L of insecticide and 188 L of larvicide were used in 2011, and 807 L of insecticide and 21 L of larvicide were used in 2012 to cover an area of 36,592 m^2^. To obtain this level of coverage, activities were repeated approximately 1,442 times in 2010, 1,510 times in 2011, and 1,430 times in 2012 (data from the PHC in Cheorwon County, Gangwon Province).

The interesting finding is that the last peak of each year was shown the similar patterns in three years, that is, increasing the malaria cases in three weeks after Chuseok (Figure 4, ↓). This duration is matched with the incubation period of vivax malaria in the liver. It was probably due to the massive interregional population movement throughout the ROK during Chuseok from high risk areas to malaria free areas, in addition increasing the activities during the night without self protection from malaria vectors. It is suggested that soldiers who leave for vacation to their hometowns should be either checked or treated for malaria just like retiring ex-soldiers from their active military service (primaquine/15 mg/14 days) [15,28,29].

To evaluate transmission of malaria in a given geographic region, many factors, including temperature, mosquito density, vector capacity, climate, rainfall, and humidity, should be considered [30]. Patient incidence alone cannot provide a complete understanding of the prevalence of malaria in the ROK because there are many factors involved, including the changes in weekly population density of mosquitoes due to environmental factors, vectorial capacity, long- and short-incubation patient ratios, symptomatic and asymptomatic patient ratios, and differences in weekly rainfall and temperatures. This study analysed only two factors: incidence and *An. sinensis* s.l. However, all factors indicated that transmission of malaria would apparently be reduced except in Incheon Metropolitan City and Gyeongnam Province. There are opportunities for the eradication of malaria in the ROK due to several efforts of the Korea CDC, Government Public Institute of Health and Environment, PHCs, and related institutes.

## Conclusions

The number of patients with vivax malaria in the ROK peaked in 2010 for a third time over two decades and then decreased in the subsequent two years. This was the third time the number of cases of malaria peaked since a trend of decreasing cases was observed in 2001. It is expected that the number of cases of malaria will continue to decrease in the future, although there may be some fluctuations. However, more intensive malaria surveillance is needed in the western part of the country where foci have re-emerged to reduce the potential for future increases in the number of malaria cases in the ROK.

## Competing interests

The authors declare that they have no competing interests.

## Authors’ contributions

TSK and HWL conceived and designed the study and contributed to the execution of the research. TSK and HWL wrote the manuscript. PYC, SKA, SKL, YJK, JSK, HSK, SHC, YKP and YSH collected the mosquitoes. HCK, WJL, SKY, JG, YS, BKN, and HSK collected the information on patients and performed analysis of the mosquito population. All authors have read and approved the final manuscript.
